# Cholera burden in Ghana: a systematic review and meta-analysis of prevalence, antimicrobial resistance and risk factors

**DOI:** 10.1093/inthealth/ihaf069

**Published:** 2025-07-21

**Authors:** Frederick Kungu, Samuel Nee-Amugie Yartey, Anastasia A Asantewaa, Eric S Donkor

**Affiliations:** Department of Medical Microbiology, University of Ghana Medical School, P. O. Box KB 4236, Accra, Ghana; Department of Medical Microbiology, University of Ghana Medical School, P. O. Box KB 4236, Accra, Ghana; Department of Medical Microbiology, University of Ghana Medical School, P. O. Box KB 4236, Accra, Ghana; Department of Medical Microbiology, University of Ghana Medical School, P. O. Box KB 4236, Accra, Ghana

**Keywords:** antimicrobial resistance, cholera, Ghana, risk factors, sanitation, *Vibrio cholerae*

## Abstract

Cholera persists in Ghana due to sanitation challenges. This systematic review aims to synthesize data on the prevalence, antimicrobial resistance, risk factors and community knowledge of cholera in Ghana. Extensive literature searches were conducted in PubMed, Scopus, ScienceDirect, Web of Science and African Journal Online. After screening, we included 33 studies, assessing their quality using the Joanna Briggs Institute checklist. Random effects meta-analysis and subgroup analysis were conducted using RStudio. The pooled prevalence of cholera was 18.42%. Based on subgroups, the highest prevalence was reported in studies that combined rectal swabs and stool samples (57.58%), involved human populations (31.79%) and were conducted in the Greater Accra–Ashanti region (64.52%). Cotrimoxazole had the highest resistance rates (75–100%) and gentamicin the lowest (1–11%). Multidrug resistance ranged between 68% and 100%. There were reports of individual resistance genes to some antibiotics (*strA, floR* and *dfrA1*). Case fatality and mortality rates were 3.40% and 2.7%, respectively. Risk factors such as eating street-vended food and proximity to refuse dumps were also reported. Cholera persists in Ghana with high drug resistance rates and regional prevalence variations. Strengthening surveillance, improving sanitation and regulating antibiotics are critical to mitigating outbreaks and resistance spread.

## Introduction

Cholera continues to affect vulnerable populations worldwide, with global estimates indicating 1.3–4 million cases annually.^1^ The disease, caused by the bacterium *Vibrio cholerae*, primarily spreads through contaminated water and food, and thrives in areas with poor sanitation.^2^ It is characterized by severe watery diarrhoea, vomiting, dehydration and life-threatening complications if not treated,^3^ leading to rapid outbreaks in settings with poor sanitation and limited access to healthcare.^4^

Africa shoulders a disproportionate cholera burden, accounting for half of global cases and 60% of deaths.^5^ Ghana exemplifies this crisis, with recurring outbreaks in urban slums and flood-prone regions where water, sanitation and hygiene (WASH) infrastructure lags.^6^ Like much of sub-Saharan Africa, Ghana's case fatality rates often exceed the World Health Organization (WHO) threshold, with children <5 y of age and displaced populations at highest risk. The country's experience mirrors broader regional challenges of vaccine shortages and weak surveillance,^7^ making cholera a persistent threat across vulnerable communities in Africa.

Despite the proven effectiveness of WASH interventions in reducing cholera in high-income countries, outbreaks persist in low- and middle-income countries such as Ghana.^6^ Recurrent outbreaks driven by overcrowding, inadequate healthcare infrastructure and antimicrobial resistance (AMR) highlight systemic challenges.^8^ For instance, during the 2014–2015 outbreak, Ghana reported approximately 29 000 cases and 243 deaths.^9^ The persistence of cholera in the country is further evidenced by the ongoing outbreak reported in October 2024,^10^ fuelled by chronically poor sanitation in most parts of the country, coupled with rapid unplanned urbanization in major cities. Compounding these issues, the clinical similarity between cholera and other diarrheal diseases sometimes delays laboratory confirmation,^11^ hindering early detection of outbreak sources and effective management. These gaps critically undermine efforts to control cholera, a preventable disease that remains a dire public health threat in Ghana and strains limited healthcare resources.

While existing studies have reported incidence of cholera in Ghana^12^,^13^ and reviewed outbreak patterns,^6^ critical gaps remain as none has consolidated data on tested cases and important parameters such as AMR trends. Therefore, this systematic review seeks to synthesize data on the prevalence of AMR, risk factors and community knowledge of cholera in Ghana, identifying gaps in current knowledge. The findings aim to directly inform Ghana's cholera control strategies, offering evidence-based solutions to mitigate future outbreaks and AMR threats.

## Methods

The Preferred Reporting Items for Systematic Reviews and Meta-Analyses (PRISMA) 2020 guidelines were followed in conducting this review. The PRISMA Checklist is included in the supplementary materials.^14^ The study protocol was registered in the PROSPERO database (CRD42025640117).

### Search strategy and study selection

Literature searches were conducted in five electronic databases: PubMed, Web of Science, ScienceDirect, Scopus and African Journals Online (AJOL). There were no time restrictions on the searches, as we included articles from the inception of each database to the search date. The searches were conducted from 7 to 13 January 2025. To retrieve relevant articles, specific keywords from the topic were used in conjunction with the Boolean operators ‘AND’ and ‘OR’ to combine similar terms and separate different ones, respectively. In all five databases, the specific string of search terms used was ‘(‘Cholera’ OR ‘*Vibrio cholerae*’) AND (‘Ghana’ OR ‘West Africa’)’.

Results from four of the databases (PubMed, Scopus, Web of Science and ScienceDirect) were downloaded as Research Information Systems (RIS) files, while articles from the AJOL database were individually downloaded. All the articles were imported into the reference manager Zotero version 7.0.11, merged into a single collection and then imported as a single RIS file into the Rayyan platform (Rayyan, Cambridge, MA, USA),^15^ which was accessed on 16 January 2025. Duplicate records were then detected and manually resolved, leaving unique articles that were further screened independently by two reviewers (FK and SNAY), based on their titles and abstracts. All unrelated articles were excluded, leaving relevant articles to be further scrutinized through full-text screening. The articles included were imported to Zotero, where their PDFs were attached for full-text screening and data extraction by the two reviewers.

### Eligibility criteria

This systematic review included peer-reviewed research articles that reported on the prevalence of cholera in Ghana, the different serotypes, and antimicrobial resistance of *V. cholerae* isolates, as well as resistance genes. Studies that assessed risk factors and knowledge of cholera were also included. Studies were excluded if they were reviews or sourced from non-peer-reviewed materials such as theses, editorials, comments and preprints. Research conducted in other African countries without Ghana-specific data were also excluded.

### Data extraction

One reviewer (FK) designed a standard table for extraction of data from the reviewed studies in Excel 365 version 2502 (Microsoft, Redmond, WA, USA), which was then discussed with two other reviewers (ESD and SNAY). Two reviewers (FK and SNAY) worked independently using the designed Excel table to extract relevant information from the studies. The extracted information included the following: author(s), year of publication, study design, geographical area of the study, sample type, number of cholera cases reported, number of cholera cases tested, number of positive cholera cases and mortality and case fatality rates (CFRs). Additionally, data were extracted on the different serogroups, serotypes and circulating genes of *V. cholerae*, along with their respective frequencies. Data on the knowledge of cholera and risk factors were also extracted from the studies. The extracted data were comprehensively and qualitatively synthesized due to heterogeneity in reported outcomes, ensuring all relevant information across studies was uniformly captured for subsequent analysis. The data were then checked by a third reviewer (ESD). All discrepancies were addressed among the three reviewers until collective agreement was reached.

### Statistical analysis

Frequency and mean calculations were conducted using Excel 365. Meta-analysis was performed using RStudio version 4.4.2 (Posit, Boston, MA, USA), which was used in calculating the prevalence of cholera from the included studies and subgroup analyses based on the study characteristics. It was also used to assess publication bias by calculating the Egger's regression and generating a funnel plot (Supplementary Figure 1). The Freeman–Tukey double arcsine transformation was used in stabilizing variances among studies and the pooled prevalences were computed using the DerSimonian–Laird method. Heterogeneity between studies was evaluated using I^2^ statistics, with values of 0%, 25%, 50% and ≥75% representing no, low, moderate and high heterogeneity, respectively. The proportion of cholera was measured by random effects meta-analysis with a confidence interval (CI) of 95%. A leave-one-out sensitivity analysis and meta-regression were performed to assess the robustness and sources of heterogeneity in our study, respectively. An α-value <0.05 was considered statistically significant.

### Quality assessment

The quality of the studies included were assessed by two reviewers (FK and SNAY) using the Joanna Briggs Institute checklist for prevalence studies.^16^ The checklist consisted of tools such as the appropriateness of the sample frame, appropriate recruitment of study participants, adequacy of sample size, detailed description of study subjects and setting, comprehensiveness of data analysis to cover the entire sample, validity of diagnostic methods, reliability of standards to measure condition, availability of appropriate statistical analysis and adequacy of the response rate. Each checklist item was scored ‘yes’, ‘no’, ‘unclear’ or ‘not applicable’. A ‘yes’ response to each question was scored 1, while ‘unclear’, ‘no’ and ‘not applicable’ were scored 0. The quality of the included studies (risk of bias) was determined as either ‘good’ (low risk; score=7–9), ‘fair’ (moderate risk; score=4–6) or ‘poor’ (high risk; score=0–3). The quality of the included studies can generally be considered good, as most of the studies (n=16) were rated ‘fair’, 15 studies were rated ‘good’ and just 2 studies were rated as ‘low’ quality (Supplementary Table 1).

## Results

### Search results

**Figure 1.**
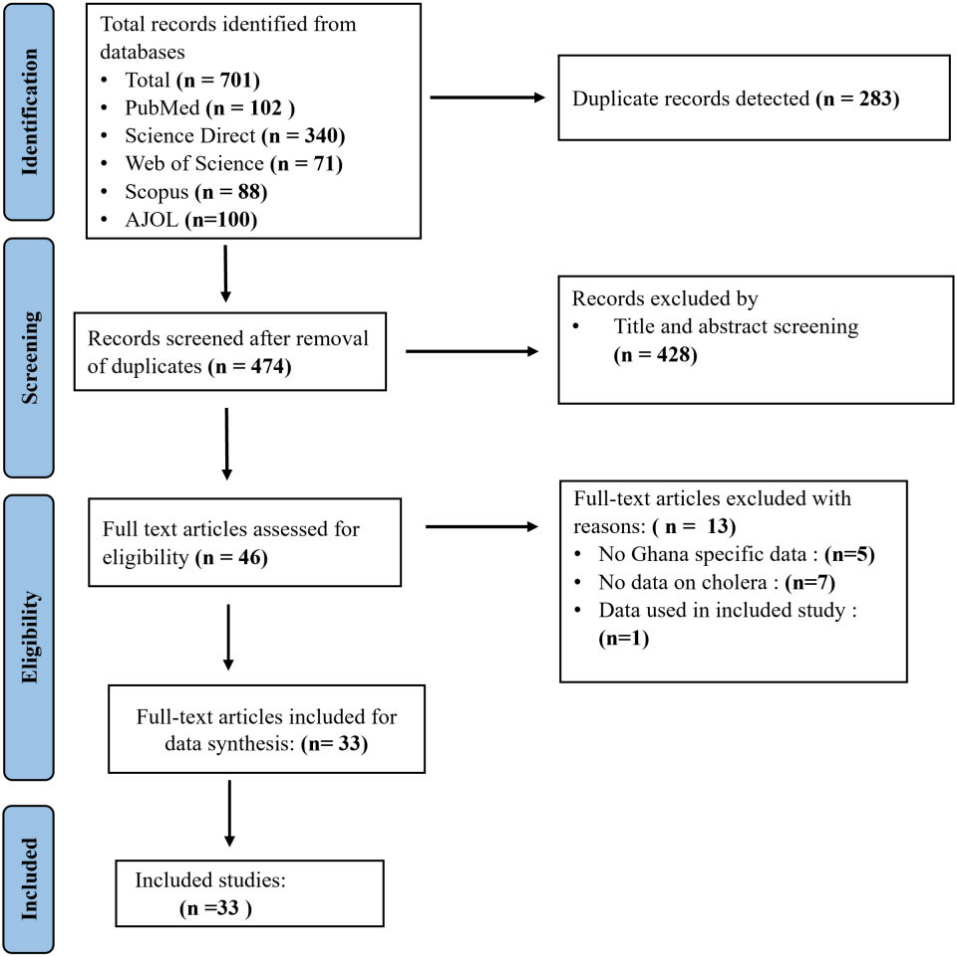
PRSIMA diagram of study selection.

A total of 710 studies were identified from the five databases: PubMed (n=102), ScienceDirect (n = 340), Web of Science (n=71), Scopus (n=88) and AJOL (n=100). Of these, 283 were duplicates, which were then resolved, leaving 474 unique articles. After title and abstract screening, 428 articles were excluded due to irrelevance to the topic. The remaining 46 articles were further scrutinized through full-text screening, of which 13 were excluded for the following reasons: generalized African or West African study with no specific data about cholera in Ghana (n=5), only mentioned cholera in their introduction or discussion with no data on cholera (n=7) and used data the same as that in another included study (n=1). This left 33 studies to be included in the systematic review (Figure 1).

### General characteristics of included studies

**Figure 2.**
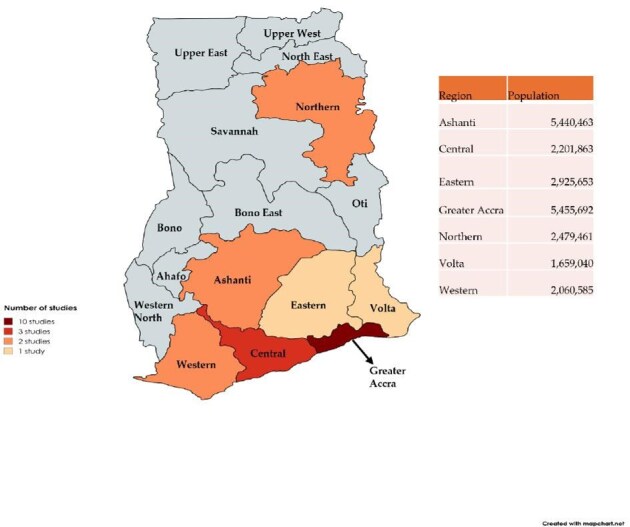
Map of Ghana showing study distribution across various regions and their respective populations. Created with MapChart^50^ and Microsoft Excel 365 version 2503.

There were 33 studies^17–49^ included in this systematic review, including studies that reported the prevalence of cholera in Ghana, antimicrobial resistance of *V. cholerae* isolates, their serotypes and the reported resistance genes. Additionally, studies that reported on risk factors and knowledge of cholera were included. The included studies covered various regions, with Greater Accra being the most frequently reported, featured in 10 studies. These included 5 individual studies focused solely on Greater Accra and 5 studies that combined Greater Accra with other regions. The Northern, Western, Central, Ashanti and Brong Ahafo regions were each reported in individual studies. Additionally, two studies covered broader areas, with one conducted across five regions and another including all regions (Figure 2). Prevalence was reported in 48% (16/33) of the included studies. Twelve studies and seven studies reported antimicrobial resistance rates and circulating genes in *V. cholerae* isolates, respectively. CFRs were reported in five studies (Table 1).

**Table 1.** Characteristics of the studies included in the meta-analysis.

Reference	Study design	Region	Population	Sample type	Cases tested, n	Positive cholera isolates, n	Risk factors
Abana et al.^17^	Cross-sectional	Greater Accra	Environment	Water	244	11	N/A
Abanyie et al.^18^	Cross-sectional	Northern	Environment	Water	1694	198	N/A
Acquah et al.^19^	Retrospective cohort	Western	Environment		61	17	N/A
Afum et al.^22^	Cross-sectional	Greater Accra	Human	Stool	772	9	N/A
Akuffo et al.^23^	Prospective cohort	Greater Accra, Northern	Human	Stool	153	1	N/A
Baah et al.^24^	Cross-sectional	Greater Accra	Animal	Meat	558	20	N/A
Danso et al.^27^	Retrospective descriptive	All regions	Human	Stool	277	168	N/A
Dzotzi et al.^28^	Prospective cross-sectional	Greater Accra	Human	Rectal swabs	218	71	N/A
Eibach et al.^29^	Retrospective longitudinal	Greater Accra, Central, Western, Eastern, Ashanti, Volta	Human	Stool	496	92	Old age is a risk factor for mortality
Enchill et al.^30^	Cross-sectional	Greater Accra	Environment	Bath towels	50	1	N/A
Feglo and Sewurah^31^	Retrospective descriptive	Greater Accra, Ashanti	Human	Rectal swabs	62	40	N/A
Issahaku et al.^32^	Descriptive case–control	Central	Human	Stool samples and rectal swabs	331	123	Visit to the cholera treatment centre, drinking pipe-borne water, drinking sachet water, eating street-vended food, washing hands, poor environmental hygiene
Noora et al.^34^	Retrospective descriptive	Brong Ahafo	Human	Stool samples and rectal swabs	199	153	N/A
Ocran and Tagoe^35^	Cross-sectional	Central	Human	Not stated	27	12	N/A
Odonkor and Addo^36^	Cross-sectional	Not stated	Environment	Water	510	6	N/A
Osei-Tutu and Anto^47^	Retrospective longitudinal	Greater Accra	Human	Not specified	27 766	61	N/A

N/A: not available.

### Pooled prevalence of cholera

**Figure 3.**
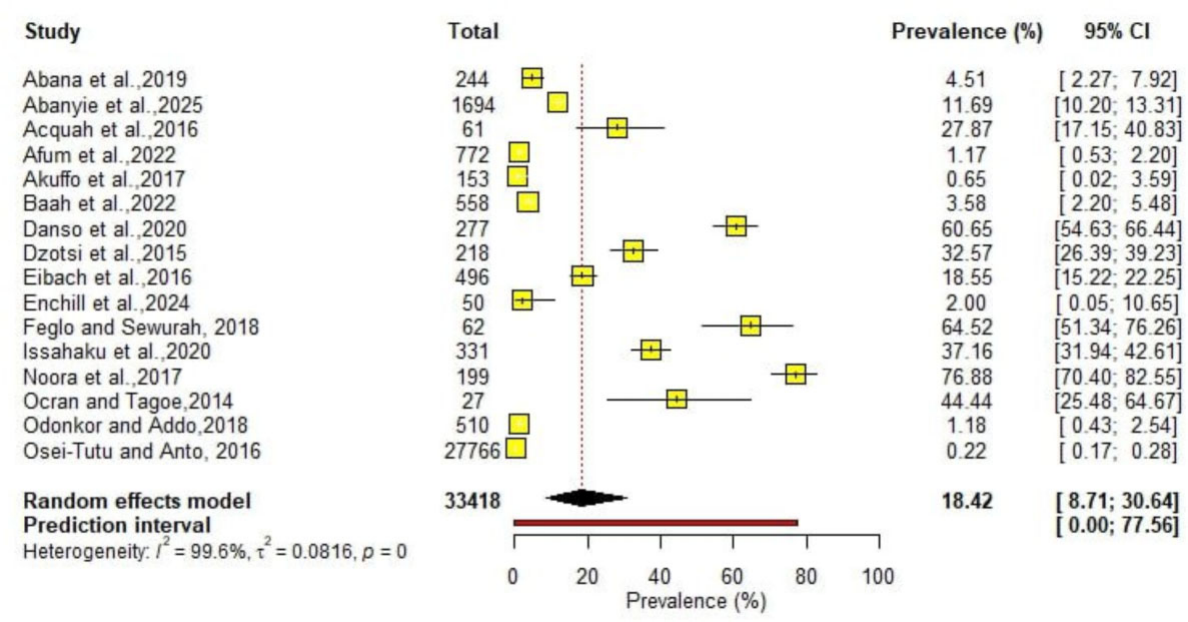
Pooled prevalence of cholera in Ghana.

Prevalence data were extracted from 16 studies.^17–19^,^22–24^,^27–32^,^34–36^,^47^ These studies reported presumptive cholera cases and specified the number of isolates that were confirmed through laboratory testing. The studies reported a total of 33 418 cases of cholera. The random effect meta-analysis of the data showed a pooled prevalence of 18.42% (95% CI 8.71 to 30.64) (Figure 3).

### Subgroup analysis based on sample type

Subgroup analysis based on sample type of the included studies reports a high prevalence in studies that took both stool samples and rectal swabs (57.58% [95% CI 19.41 to 91.08]), as compared with individual studies that used rectal swabs (47.92% [95% CI 18.54 to 78.10]) and stool samples (14.11% [95% CI 0.06 to 44.40]) alone. A prevalence of 4.99% (95% CI 0.34 to 14.31) was observed in water samples alone, while studies with non-specified sample types reported a relatively significant prevalence of 17.59% (95% CI 0.00 to 61.50). The lowest prevalences were reported in sample types reported in just one study. These included meat samples at 3.58% (95% CI 2.18 to 5.30) and bath towels at 2.00% (95% CI 0.00 to 8.39) (Supplementary Figure 2).

### Subgroup analysis based on population

**Figure 4.**
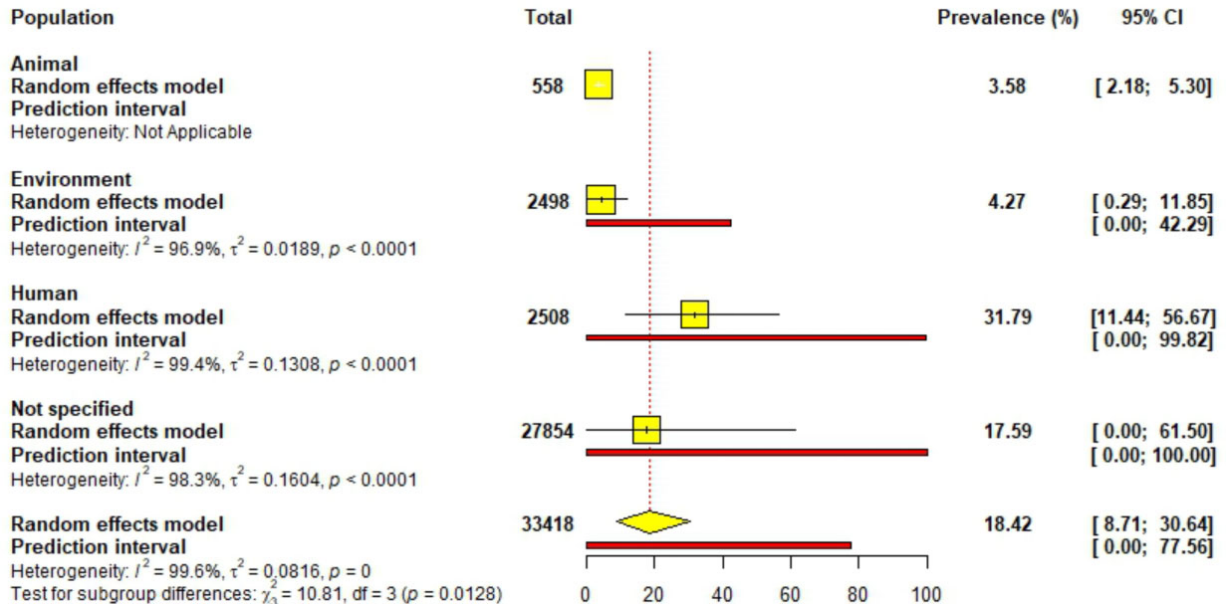
Subgroup analysis based on population type.

Classifying the samples based on the population type, the subgroup analysis showed the highest prevalence in human studies at 31.79% (95% CI 11.4 to 56.67), followed by environmental studies at 4.27 (95% CI 0.29 to 11.85) and animal studies at 3.58% (95% CI 2.18 to 5.30). Studies with an unspecified population type showed a high prevalence of 17.59% (95% CI 0.00 to 61.50) (Figure 4).

### Subgroup analysis based on region

In the subgroup analysis of cholera prevalence in Ghana by region, the highest rates were observed in combined data from Greater Accra and Ashanti (64.52% [95% CI 52.13 to 76.02]), as well as studies conducted across all regions (60.65% [95% CI 54.82 to 66.33]). This was followed by the Central region with a prevalence of 37.52% (95% CI 32.48 to 42.69). Greater Accra alone showed a lower prevalence of 4.68% (95% CI 0.65 to 11.71) but extreme heterogeneity. The combined Greater Accra–Northern data reported minimal cases (0.65% [95% CI 0.00 to 2.79]). The Northern (11.69% [95% CI 10.20 to 1.26]) and Western (27.87% [95% CI 17.25 to 39.87]) regions showed intermediate prevalence (Supplementary Figure 3).

### Antimicrobial resistance and genetic determinants

#### Antimicrobial resistance

Antimicrobial resistance was reported in 10 studies,^17^,^24^,^27–29^,^31^,^33^,^41^,^48^,^51^ with sulfamethoxazole/trimethoprim (8 studies), ampicillin (8 studies) and nalidixic acid (6 studies) being the most reported. Cefuroxime, ceftriaxone, tetracycline and cefotaxime were reported in three studies each, while ciprofloxacin, gentamicin, ceftazidime, amikacin and erythromycin reported in two studies each. Multidrug resistance (MDR) was reported in three studies. There were wide variations observed in resistance to various antibiotics. Ampicillin (18–100%) and sulfamethoxazole/trimethoprim (25–100%) showed very high resistance. Nalidixic acid (7–100%), ciprofloxacin (17–98.4%) and ceftriaxone (2–75%) exhibited highly variable resistance. Erythromycin (58–92.5%) also showed high resistance. Cefuroxime (4–26%), ceftazidime (3–11%), gentamicin (1–11%) and amikacin (11–16%) had relatively lower resistance. Alarming levels of MDR (48–100%) were also reported (Supplementary Table 2).

#### Resistance genes and other genetic determinants

**Figure 5.**
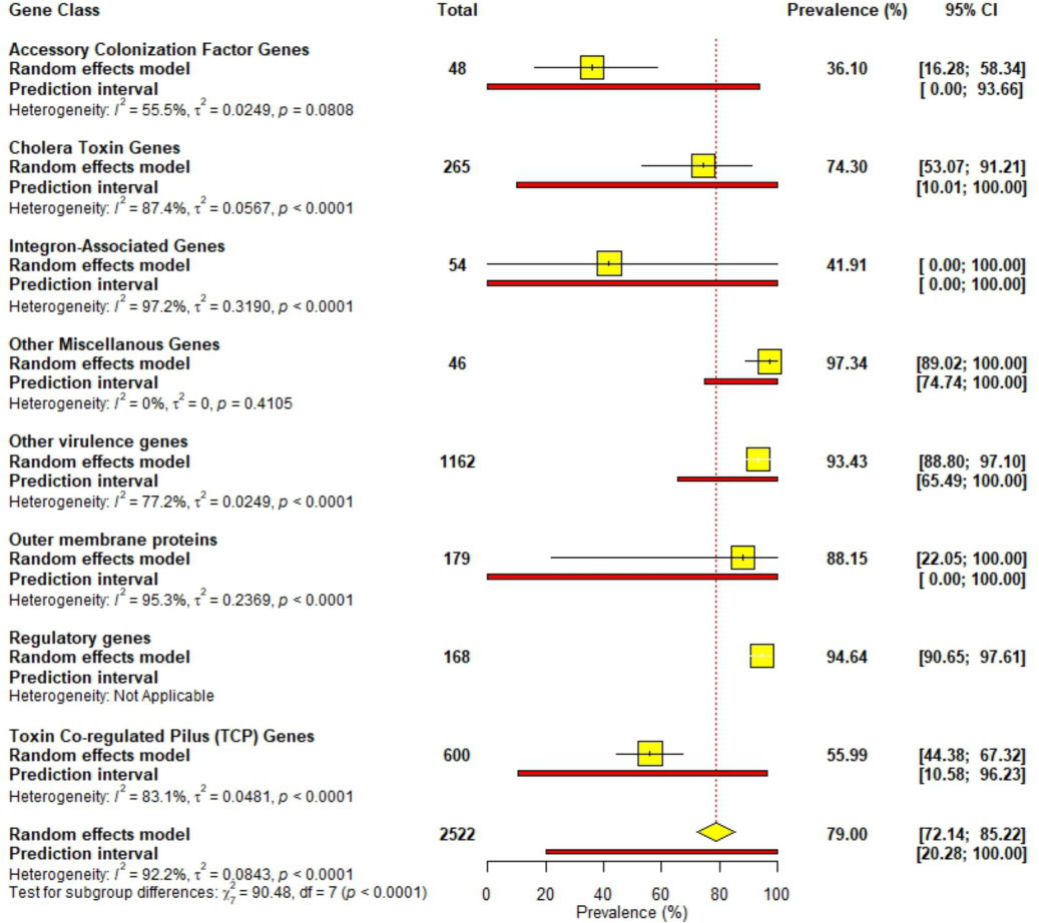
Prevalence of circulating gene classes.

The meta-analysis revealed that regulatory genes (94.64% [95% CI 90.65 to 97.61]) and miscellaneous genes (97.34% [95% CI 89.02 to 100.00]) were most prevalent in *V. cholerae* isolates, followed by outer membrane proteins (88.15% [95% CI 22.05 to 100.00]) and toxin co-regulated pilus genes (55.99% [95% CI 44.38 to 67.32]). Accessory colonization factor genes (36.10% [95% CI 16.28 to 58.32]) and integron-associated genes (41.91% [95% CI 0.00 to 100]) showed the lowest prevalence, with the latter exhibiting extremely wide CIs (Figure 5).

### Circulating serotypes of *V. cholerae* in Ghana

**Figure 6.**
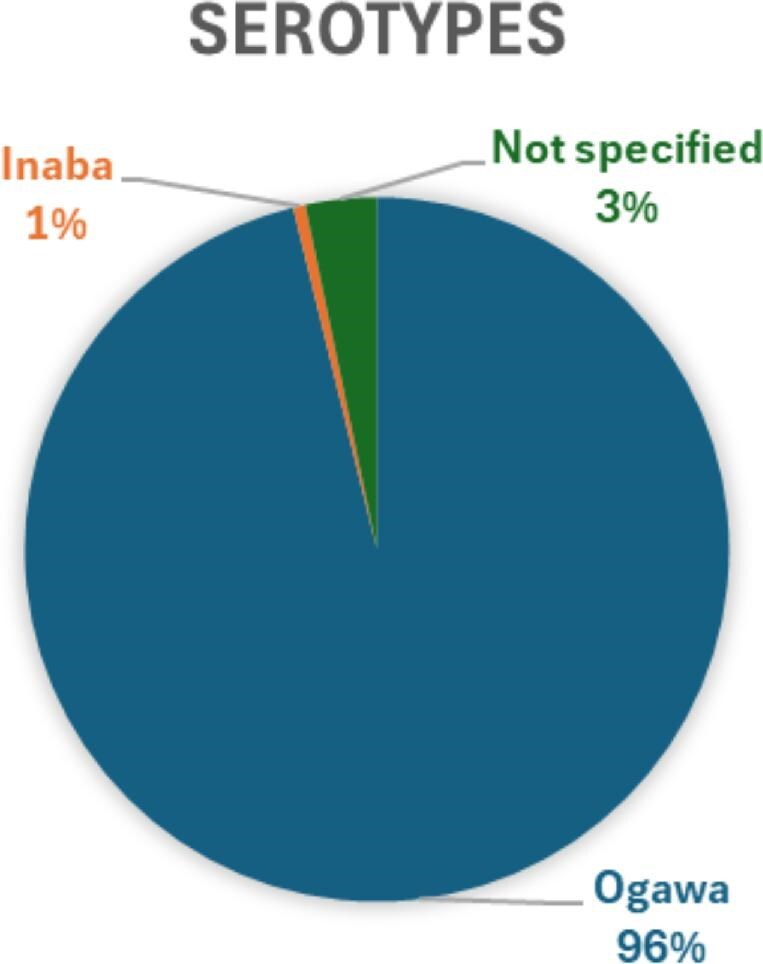
Distribution of *V. cholerae* serotypes.

Among the studies included, 33% (11/33) reported on the *V. cholerae* serogroups and serotypes identified. Two serogroups were documented, with the O1 serogroup being the most prevalent at 96.7% (682/705). Only 3.2% (23/705) were classified as non-O1/non-O139. Two serotypes were identified: Ogawa, with 691 isolates, and Inaba, with 14 isolates. Serotypes were not specified for isolates belonging to the non-O1/non-O139 serogroup (Figure 6).

### Knowledge of cholera

Three of the included studies assessed the knowledge of individuals in the community, involving a total of 1002 individuals within the Ashanti, Greater Accra and Eastern regions. One study assessed individuals from high school while the others engaged members of the general community. Overall, participants were largely at the high school education level. Knowledge of the causes of cholera was generally good, with 59% of respondents demonstrating awareness. In another study by Tutu et al.,^49^ conducted in cholera-endemic communities, high levels of knowledge were reported on cholera risk factors (92.6), food safety practices (85.78%) and personal hygiene practices (87.2%). Participants also showed good awareness of environmental risk factors (67.2%) and signs of the disease (65.2%) (Supplementary Table 3).

### Risk factors

Risk factors were reported in nine studies.^21^,^29^,^32^,^39^,^43–46^ The studies were across the Greater Accra, Central, Eastern, Western, Volta and Ashanti regions. According to Adjei et al.,^21^ cholera outbreaks peaked in March 2011 and April 2012. Key risk factors included environmental and sanitation conditions, such as proximity to refuse dumps, high refuse density, poor sanitation practices and poor environmental hygiene. As a major factor in the transmission of cholera, water and food sources played a crucial role, including drinking from streams, consuming street-vended food and eating *banku* and *fufu* (local staple foods mostly eaten with soups). However, drinking pipe-borne water or sachet water was protective.

Population density and urbanization were also key risk factors, with slum areas, overcrowding and high population density and neighbourhood disorder increasing transmission. Case clustering within 1 km and household interactions were also significant factors that facilitated spread, while proximity to index cases heightened the risk. Older adults were reported to face high mortality rates and visiting cholera treatment centres was associated with infection (Supplementary Table 4).

### CFRs and mortality rates

CFRs and mortality rates were reported in six studies.^19^,^21^,^28^,^37–39^ The CFR ranged from 0.18% (95% CI 0.00 to 2.20) to 18.80% (95% CI 7.21 to 36.44), with a mean of 3.40% (95% CI 3.35 to 3.44). The mortality rate ranged from 0% (95% CI 0.00 to 2.20) to 18.75% (95% CI 8.89 to 35.31) and the mean mortality rate was 2.76% (95% CI 0.00 to 7.40) (Table 2).

**Table 2.** Case fatality and mortality rates

Reference	CFR, %	Sample size, n	Mortality, n	Mortality rate
Acquah et al.^19^	5.90	61	0	0
Adjei et al.^21^	0.95	1056	10	0.95
Dzotzi et al.^28^	1.40	218	4	1.83
Ohene et al.^37^	1.20	422	5	1.18
Ohene et al.^37^	18.80	32	6	18.75
Ohene-Adjei et al.^38^	0.30	4023	12	0.3
Ohene-Adjei et al.^38^	0.50	12 255	64	0.52
Ohene-Adjei et al.^38^	1.40	3339	45	1.35
Opare et al.^39^	0.18	136	0	0

### Meta-regression

Our meta-regression analysis revealed no statistically significant associations between sample type and cholera prevalence, with all comparisons yielding p-values >0.05. Meat samples (estimate=0.02, p=0.97), rectal swabs (estimate=0.44, p=0.53), stool samples (estimate=−0.06, p=0.93) and water samples (estimate=−0.15, p=0.82) showed negligible effects. Similarly, regional comparisons indicated no significant differences in cholera prevalence. The Greater Accra region (estimate=−0.78, p=0.26) and combined regions (e.g. Greater Accra/Ashanti: estimate=−0.46, p=0.64) exhibited trends toward lower prevalence, although these were not statistically significant. The intercept (estimate=0.95, p=0.26) suggested a baseline cholera detection rate, but with substantial uncertainty (95% CI −0.71 to 2.62). Overall, this analysis suggests that either cholera is spread similarly across regions or sample types or, alternatively, there may be insufficient data to identify true differences in cholera distribution within Ghana (Supplementary Table 5).

### Sensitivity analysis

**Figure 7.**
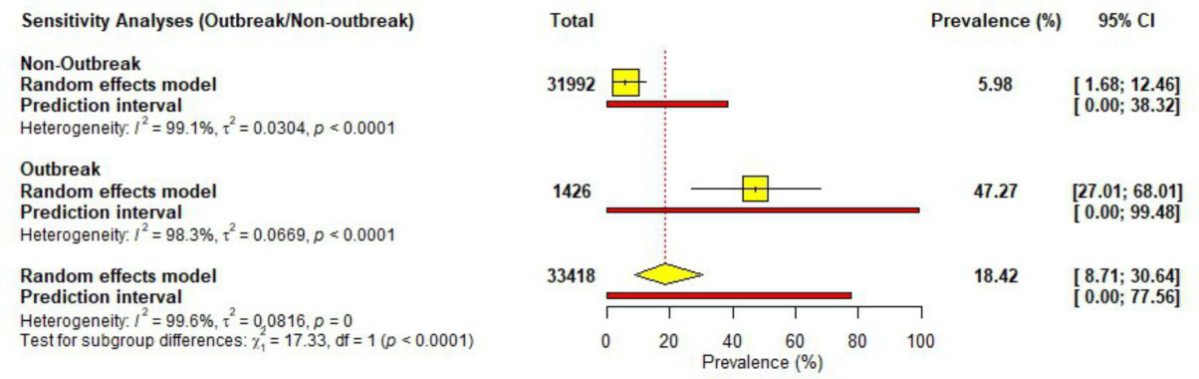
Sensitivity analysis between outbreak and non-outbreak studies.

To evaluate the impact of each included study on the pooled prevalence of cholera observed in the meta-analysis, a leave-one-out sensitivity analysis was performed. The point estimates ranged from 15.20% to 20.59%, with consistently overlapping CIs, suggesting that there were no statistically significant variations in the point estimates. Although there were some observed differences, the study selection can be considered robust, as the individual prevalence estimates were closely aligned with the pooled prevalence of 18.42% (Supplementary Table 6). Additionally, a sensitivity analysis comparing outbreak and non-outbreak studies was also conducted, revealing substantial differences in the pooled prevalence of cholera. Outbreak-related studies reported a markedly higher prevalence of 47.27% (95% CI 27.01 to 68.01), whereas non-outbreak studies showed a significantly lower prevalence of 5.98% (95% CI 1.68 to 12.46) (Figure 7).

## Discussion

Recurrent cholera outbreaks in Ghana reflect the persistent challenges in addressing the behavioural and environmental drivers that undermine effective WASH compliance, particularly in high-risk urban and coastal communities. These structural and knowledge gaps directly contribute to the enduring high prevalence of cholera and other food and waterborne diseases, perpetuating cycles of preventable transmission. In this systematic review, we report a pooled prevalence of cholera of 18.42%, indicating the substantial burden of the disease in the country.

Our meta-analysis, limited to laboratory-confirmed cases, suggests that this figure likely underestimates the true disease burden. Most outbreak investigations in Ghana rely on clinical definitions for surveillance once an initial toxigenic *V. cholerae* isolate has been confirmed.^52^ This disparity is evident in the ongoing 2024–2025 outbreak, where only 11% of 4951 suspected cases had laboratory confirmation as of February 2025.^53^ Despite the significant pooled prevalence reported, our sensitivity analysis comparing outbreak vs non-outbreak suggests that the overall pooled prevalence of cholera in Ghana may be influenced by the inclusion of outbreak-related studies, which reported substantially higher prevalence rates (Figure 7). This indicates that the total pooled estimate may overrepresent the cholera burden during non-outbreak periods, emphasizing the need to interpret the overall prevalence in the context of outbreak dynamics. Additionally, only 7 (44%) of the 16 regions were represented in the included studies, with most conducted in the southern part of the country (Figure 6), limiting the scope of our results. This aligns with the findings of Donkor and Namaitijiang,^54^ who report that the Greater Accra region accounts for more than half of the cholera cases in Ghana.

The subgroup analysis based on sample types showed that studies that combined both stool samples and rectal swabs reported higher prevalence rates, implying the enhanced sensitivity of dual sampling methods in detecting *V. cholerae—*likely due to increased bacterial load capture (stool) and early-stage infection identification (swabs).^55^ Based on the population type, human samples were more prevalent, as they were the most included, followed by environmental studies, with animal populations being the least, as there was only one study included.

In this systematic review, we report a mean CFR of 3.40%, which is significantly higher than the 0.9% reported in the 2014–2015 outbreak,^56^ and more than triple the minimum acceptance threshold of 1%.^57^ However, the small sample size (five studies) from which our analysis was conducted, coupled with the different study periods, may explain the higher CFR observed in this review. In comparison, our study reports a lower CFR than that reported in Nigeria (4.87%).^58^ In Ethiopia, a retrospective analysis spanning two decades reported a lower CFR (1.10%), however with a sample size 10 times that analysed in our study.^59^ The reported mortality rate of 2.76% calculated in our study understates the threat of the ongoing outbreak, which already reports a mortality rate of 8.92%.^10^ This may be due to factors such as the underreporting of cases and deaths in resource-limited settings and delayed healthcare-seeking behaviour, further suggesting that the true burden of the disease may be higher than estimated. The variation between our findings and national surveillance data highlights the need for ongoing monitoring and more comprehensive studies to reconcile these differences, as factors such as outbreak intensity and system responses may influence mortality estimates over time. According to a study by El Bushra et al.,^60^ the major factor in reducing the CFR was access to medical services.

The major risk factors identified in our study were linked to environmental and sanitation concerns. Factors such as proximity to refuse dumps and high population densities in slum areas foster disease transmission. Additionally, consuming street-vended food, particularly soupy dishes, was also a major contributor to spread of the disease. Drinking water from streams was a high-risk factor. Safe drinking water pipelines that passed through open drainages were prone to contamination and could also foster spread of the disease.^32^

Over the years, antimicrobial resistance has been reported in *V. cholerae* isolates worldwide,^61^ creating an extra hurdle in managing cholera. In our study, the most resistance was reported in sulfamethoxazole/trimethoprim, ampicillin and nalidixic acid, respectively. Similarly, Mohammed et al.,^62^ in their systematic review, reported sulfamethoxazole/trimethoprim, ampicillin and chloramphenicol as the antibiotics with the most reported resistance in their included studies. The highest resistance rates were reported in sulfamethoxazole/trimethoprim and nalidixic acid, similar to findings by Yuan et al.^63^ In contrast, there was increasing resistance in ampicillin, ciprofloxacin, ceftriaxone and erythromycin as opposed to the decrease in these antibiotics reported by Yuan et al.^63^ Ceftazidime, cefuroxime, gentamicin and amikacin reported the least resistance (Supplementary Table 2), similar to recent findings.^64^,^65^ MDR was also reported in a few studies, with resistance rates up to 100% in some studies, in line with recent reports.^61^,^66^ Regulatory genes were the most prevalent, as they play crucial roles in the virulence and environmental survival of *V. cholerae* isolates.^67^ There were also resistance genes (*strA, strB, catB5, flor2, sul2* and *dfrA1*)^41^ and genetic elements (SXT, class I and II integrons)^51^,^68^ that are not resistance genes themselves but play crucial roles in expressing AMR genes.^69^,^70^ The detection of resistance genes such as *strA* and *floR* in *V. cholerae* isolates traditionally suggests resistance to streptomycin and chloramphenicol, respectively.^71^ However, it may also suggest the presence of integrons or plasmids harbouring multiple resistance genes,^71^ including those conferring resistance to sulfamethoxazole. The co-occurrence of *strA* with *sul* genes on mobile genetic elements has been documented in *V. cholerae*,^72^ facilitating the horizontal spread of resistance traits. This genetic linkage likely contributes to the high resistance to sulfamethoxazole reported in most studies. From a clinical and public standpoint, these findings emphasize the importance of molecular surveillance of resistance determinants in cholera pathogens, not only to understand AMR trends, but also to guide appropriate antimicrobial containment strategies during outbreaks.

Generally, there was good knowledge of the causes, food safety practices and risk factors associated with cholera. This contrasts with the findings of Tutu et al.,^49^ who report the existence of substantial gaps in knowledge of the environmental risk factors associated with cholera. These knowledge gaps, as well as other factors such as urban slums, climate impacts such as flooding due to poor drainage systems and poor waste management and healthcare access gaps, enhance the persistence of cholera in Ghana.^73^

Ghana has implemented several measures to control infectious diseases such as cholera. For instance, in 2016, the Ministry of Health of Ghana, in collaboration with the WHO, USAID/Global Communities and UNICEF, launched a national cholera prevention campaign aimed at eliminating cholera by engaging at-risk communities and promoting preventive practices.^74^ Moreover, strengthening existing systems such as integrated disease surveillance and response (ISDR)^75^ and WASH policies has also been a key strategy to enable early outbreak detection and management and to improve access to clean water and sanitation. These systems, in addition to the introduction of community-led education programs and public education and sanitation campaigns are effective approaches to improve hygiene practices and healthcare-seeking behaviour. Additionally, the oral cholera vaccine has been introduced in cholera-endemic regions,^53^ with plans for a nationwide extension of the initiative. However, these measures are hindered by numerous challenges, including urban–rural disparities, inconsistent infrastructure, overcrowding, persistence of open defecation and contaminated water sources. Limited funding and logistical hurdles slow the progress of the fight against cholera.

### Strengths and limitations

This systematic review brings together available data on cholera in Ghana, providing a broad overview of its epidemiology, risk factors, resistance patterns and knowledge-related gaps. However, the findings are constrained by heterogeneity in study designs, underrepresentation of all regions and potential underreporting. Also, the small number of included studies may also influence prevalence rates and skew other statistical values due to the unavailability of data. Our meta-regression analysis reports no strong evidence that where samples were taken (region) or what kind of sample was tested had any significant effect on cholera prevalence. This could be due to the lack of data to detect real differences and the concentration of studies in urban areas, particularly in the Greater Accra region—with 10 of the 16 prevalence studies conducted there. This disproportionately skews the overall prevalence rate, underrepresenting other parts of the country. Our funnel plot asymmetry and Egger's test suggest potential publication bias. This may be due to the variability in study designs, study periods and sample sizes of the included studies. Additionally, although non-English reports were not encountered and most grey literature was inaccessible, their absence may have limited data diversity and contributed to an overestimation of prevalence, particularly during outbreak years. Despite these limitations, this systematic review offers critical insights to inform cholera control policies in Ghana.

## Conclusions and future recommendations

This systematic review and meta-analysis addressed the critical problem of assessing a holistic overview of cholera in Ghana, focusing on reporting prevalence and mortality rates, antimicrobial resistance and the circulating resistance genes, risk factors and community knowledge gaps associated with the disease. Our findings report a high prevalence of cholera, with resistance of *V. cholerae* isolates to key antibiotics and the presence of some resistance genes. We also report high environmental risk factors, but good knowledge on the causes of cholera and its risk factors. These findings underline the urgent need for policymakers and development institutions to integrate cholera management and surveillance into developmental strategies. The high level of antibiotics resistance observed also calls for increased awareness and stronger antimicrobial stewardship efforts. This study concedes limitations such as the small number of studies for statistical analysis and underrepresentation of some regions. Addressing data gaps through routine reporting and improved access to grey literature could enhance national cholera estimates. Future research should explore gender- and age-related differences in cholera prevalence and outcomes, as well as socio-economic and cultural factors that may drive transmission disparities. Additionally, genomic studies should be prioritized to track AMR evolution over time. In conclusion, cholera is still a major health threat in Ghana, demanding coordinated multisectoral efforts to reduce its burden.

## Supplementary Material

ihaf069_Supplemental_Files

## Data Availability

All data relevant to the study are included in the article and its supplementary materials.
