# Supplementary material for: Cholera burden in Ghana: a systematic review and meta-analysis of prevalence, antimicrobial resistance and risk factors
Source: Int Health. 2025 Jul 21;17(6):869–80. doi: 10.1093/inthealth/ihaf069 (PMC12585563; doi:10.1093/inthealth/ihaf069)
Supplement: ihaf069_Supplemental_Files [file ihaf069_supplemental_files.zip › Cholera in Ghana_Supplementary.docx]

The Persistent Cholera Burden in Ghana: A Systematic Review and Meta-Analysis of Prevalence, Antimicrobial Resistance, and Transmission Risks
Frederick Kungu^1^, Samuel Nee-Amugie Yartey^1^, Anastasia A. Asantewaa^1^ and Eric S. Donkor^1*^

1. Department of Medical Microbiology, University of Ghana Medical School, Accra, P. O. Box KB 4236, Ghana.

* Correspondence: Eric S. Donkor, email: [esampane-donkor@ug.edu.gh](mailto:esampane-donkor@ug.edu.gh)
**Supplementary Materials**

Supplementary Figure 1. Funnel plot of study selection


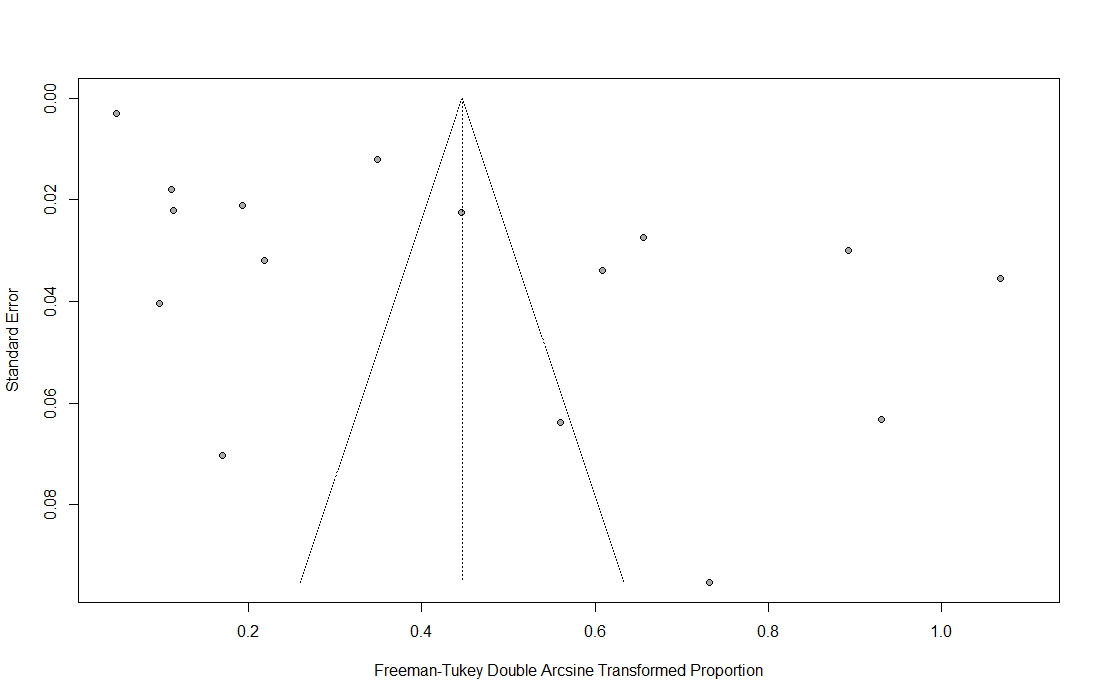


The p-value of the Egger’s Test is 0.0006 (indicating a possible publication bias)

Supplementary Figure 2. Subgroup analysis based on sample type


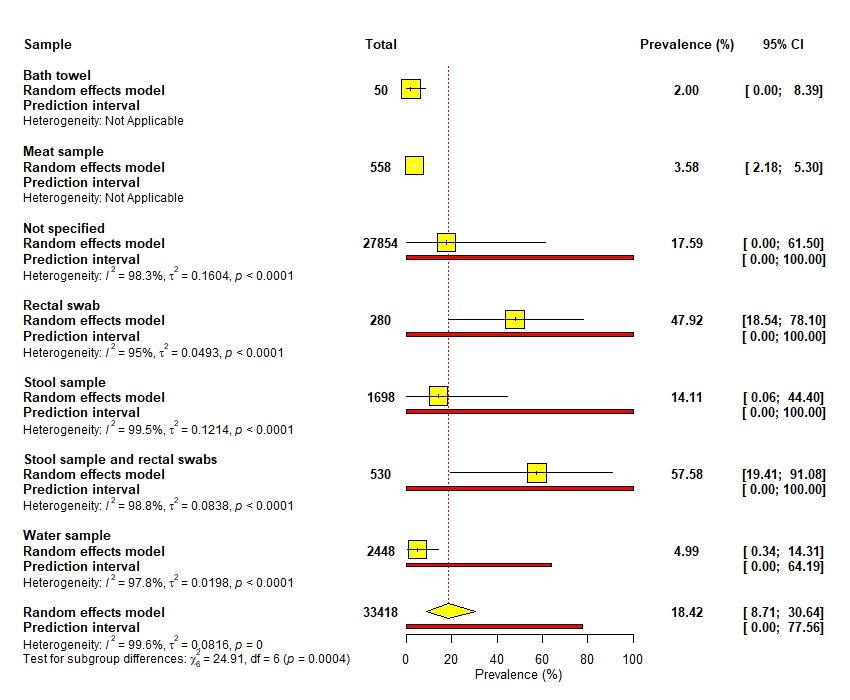


Supplementary Figure 3. Subgroup analysis based on regions


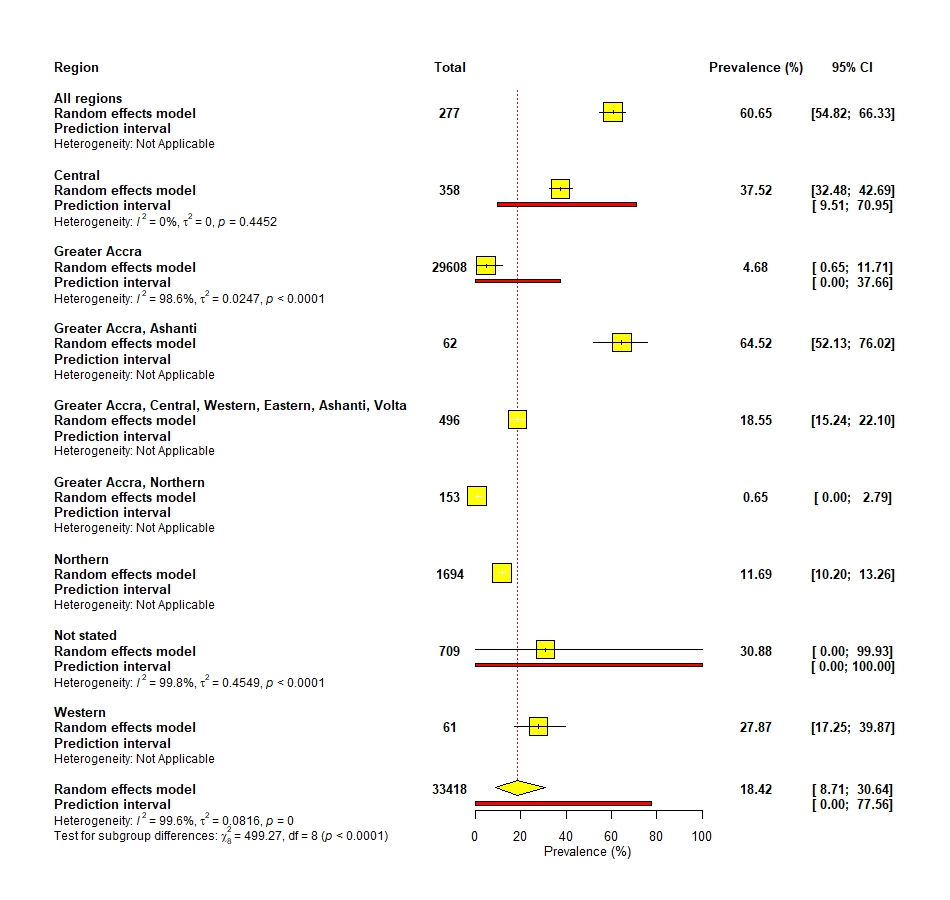


Supplementary Table 1. Quality assessment of included study based on the JBI checklist for prevalence studies.

| Study ID | Was the sample frame appropriate to address the target population? | Were study participants sampled in an appropriate way | Was the sample size adequate | Were the study subjects and the setting described in detail | Was the data analysis conducted with sufficient coverage of the identified sample | Were valid methods used for the identification of the condition | Was the condition measured in a standard, reliable way for all participants | Was there appropriate statistical analysis | Was the response rate adequate, and if not, was the low response rate managed appropriately | Total score | Remark |
| --- | --- | --- | --- | --- | --- | --- | --- | --- | --- | --- | --- |
| Abana et al.,2021 | 1 | N/A | 1 | 1 | 1 | 1 | N/A | 1 | N/A | 6 | Fair |
| Abanyie et al.,2025 | 1 | 1 | 1 | 1 | 1 | N/A | N/A | 1 | 1 | 7 | Good |
| Acquah et al.,2016 | 1 | 1 | 1 | 1 | 1 | 0 | 0 | 1 | 0 | 6 | Fair |
| Adade et al.,2022 | 0 | N/A | 0 | 0 | 1 | 1 | N/A | 0 | N/A | 2 | Poor |
| Adjei et al.,2017 | 1 | 1 | 1 | 1 | 0 | N/A | N/A | 0 | 1 | 5 | Fair |
| Afum et al.,2022 | 1 | 1 | 1 | 1 | 1 | 1 | 1 | 1 | 1 | 9 | Good |
| Akuffo et al.,2017 | 1 | 1 | 1 | 1 | 1 | 1 | 1 | 0 | 1 | 8 | Good |
| Baah et al.,2022 | 1 | 1 | 1 | 1 | 1 | 1 | 1 | 1 | 1 | 9 | Good |
| Boateng et al.,2022 | 1 | 1 | 1 | 1 | 1 | N/A | N/A | 1 | 0 | 6 | Fair |
| Codjoe et al.,2021 | 1 | 1 | 0 | 1 | 1 | N/A | N/A | 1 | 1 | 6 | Fair |
| Danso et al.,2020 | 1 | 1 | 1 | 1 | 1 | 1 | 1 | 0 | 1 | 8 | Good |
| Dzotsi et al.,2015 | 1 | 1 | 1 | 1 | 1 | 1 | 1 | 0 | 1 | 8 | Good |
| Eibach et al.,2016 | 1 | 1 | 1 | 1 | 1 | 1 | 1 | 0 | 1 | 8 | Good |
| Enchill et al., 2024 | 1 | 1 | 1 | 1 | 1 | 1 | 1 | 1 | 1 | 9 | Good |
| Feglo and Sewurah, 2018 | 1 | 1 | 1 | 1 | 1 | 1 | 1 | 1 | 1 | 9 | Good |
| Issahaku et al.,2020 | 1 | 1 | 1 | 1 | 1 | 1 | 1 | 1 | 1 | 9 | Good |
| Newman et al.,2004 | 1 | 0 | 0 | 0 | 1 | 1 | 1 | 0 | 1 | 5 | Fair |
| Noora et al.,2017 | 1 | 1 | 1 | 1 | 1 | 0 | 1 | 0 | 0 | 6 | Fair |
| Ocran and Tagoe,2014 | 1 | 1 | 1 | 0 | 1 | N/A | 1 | 1 | 0 | 6 | Fair |
| Odonkor and Addo,2018 | 1 | 1 | 1 | 1 | 1 | 1 | 1 | 0 | 1 | 8 | Good |
| Ohene et al.,2016 | 1 | 1 | 1 | 1 | 1 | N/A | N/A | 0 | 1 | 6 | Fair |
| Ohene-Adjei et al.,2017 | 1 | 1 | 1 | 1 | 1 | N/A | 1 | 1 | 1 | 8 | Good |
| Opare et al.,2012 | 1 | 1 | 0 | 1 | 1 | 0 | 0 | 1 | 1 | 6 | Fair |
| Opintan et al.,2008 | 1 | 0 | 0 | 0 | 1 | 1 | 1 | 0 | 1 | 5 | Fair |
| Opintan et al.,2021 | 1 | 1 | 1 | 1 | 1 | 1 | 1 | 0 | 1 | 8 | Good |
| Osei and Duker, 2008 | 1 | N/A | 1 | 1 | 1 | N/A | 1 | 1 | 0 | 6 | Fair |
| Osei and Duker, 2008 | 1 | N/A | 1 | 1 | 1 | N/A | 1 | 1 | 1 | 7 | Good |
| Osei et al.,2011 | 1 | N/A | 1 | 1 | 1 | N/A | 1 | 1 | 0 | 6 | Fair |
| Osei et al.,2012 | 1 | N/A | 1 | 1 | 1 | N/A | 1 | 0 | 1 | 6 | Fair |
| Osei and Stein,2018 | 1 | N/A | 1 | 1 | 1 | N/A | 1 | 1 | 0 | 6 | Fair |
| Osei-Tutu and Anto, 2016 | 1 | N/A | 1 | 1 | 1 | N/A | 1 | 1 | 0 | 6 | Fair |
| Thompson et al.,2011 | 1 | N/A | 0 | 0 | 0 | 0 | 1 | 0 | 1 | 3 | Poor |
| Tutu et al.,2019 | 1 | 1 | 1 | 1 | 1 | N/A | 1 | 1 | 1 | 8 | Good |

Yes- 1, No/ Unclear/ Not Applicable -0.

**Scale**

Good (7 – 9), Low risk of bias,

Fair (4 – 6), Moderate risk of bias,

Poor (0 – 3), High risk of bias

Supplementary Table 2. Antimicrobial resistance rates reported in some included studies

| Study ID | AMP | SXT | CXM | CHL | NA | CTR | TET | GEN | CIP | CTX | CAZ | AK | ERY | MDR |
| --- | --- | --- | --- | --- | --- | --- | --- | --- | --- | --- | --- | --- | --- | --- |
| Abana et al.,2021 | - | - | - | - | 72.50% | - | - | - | - | - | - | - | 92.50% | - |
| Baah et al.,2022 | 100% | 25% | - | - | - | - | - | - | - | - | - | - | - | - |
| Danso et al.,2020 | 67%-72% | 75%-92% | 0%-4% | 29%-33% | 25%-96% | 2%-8% | 11%-13% | 1%-2% | 17%-21% | 3%-6% | 3%-11% | 11%-16% | 58%-69% | - |
| Dzotsi et al.,2015 | 100% | 100% | - | 100% | 100% | - | - | - | - | - | - | - | - | - |
| Eibach et al.,2016 (2011) | - | 100% | - | - | - | - | - | - | - | - | - | - | - | - |
| Eibach et al.,2016 (2012) | 91.70% | 83% | - | - | 91.70% | - | - | - | 91.70% | - | - | - | - | - |
| Eibach et al.,2016 (2013) | 95.20% | 96.80% | - | - | 100% | - | - | - | 98.40% | - | - | - | - | - |
| Feglo and Sewurah, 2018 | 90% | 95% | - | - | - | 75% | - | - | - | - | - | - | - | 67.50% |
| Newman et al.,2004 | 24% | 100% | 26% | 4% | 7% | - | 7% | - | - | 26% | - | - | - | - |
| Opintan et al.,2008 | 37% | 96% | 22% | 19% | 37% | 22% | 11% | 11% | - | 15% | 11% | 11% | - | 48% |
| Opintan et al.,2021 | - | - | - | - | - | - | - | - | - | - | - | - | - | 100% |
| Thompson et al.,2011 | 18% | 59.10% | - | - | - | - | - | - | - | - | - | - | - | - |

AMP - Ampicillin; SXT – Sulfamethoxazole/Trimethoprim; CXM – Cefuroxime; COT – Cotrimoxazole; CHL – Chloramphenicol; NA – Nalidixic acid; CTR – Ceftriaxone; TET – Tetracycline; GEN – Gentamicin; CIP – Ciprofloxacin; CTX – Cefotaxime; CAZ – Ceftazidime; AK – Amikacin; ERY – Erythromycin; MDR – Multi-drug resistance

Supplementary Table 3. Knowledge of cholera

| Study ID | Study D | Region | Setting | Sample size | Education level | Knowledge (Causes) | Summary of findings |
| --- | --- | --- | --- | --- | --- | --- | --- |
| Boateng et al.,2022 | cross-sectional | Ashanti | School | 391 | 100% high school | 78% | Good knowledge of cholera causes |
| Codjoe et al.,2021 |  | Eastern | Community members | 210 | 42.60% high school | 20% | There was fair knowledge on public health threats that were likely to result from vending operations |
| Tutu et al.,2019 | cross-sectional | Greater Accra | Community members | 401 |  | 79% | Generally there is high knowledge on cholera risk factors (92.6) and personal hygiene practices (87.2%), there is good knowledge on the environmental risk factors of cholera (67.2%) and the signs of cholera (65.2%), there is high knowledge on food safety practices (85.78%) |

Supplementary Table 4. Risk factors associated with cholera

| Study ID | Study Design | Region | Setting | Sampled Population | Risk factors |
| --- | --- | --- | --- | --- | --- |
| Adjei et al.,2017 | cross-sectional | Greater Accra | Environment | Community members | Cholera peaked in March and April of 2011 and 2012 respectively |
| Opare et al.,2012 | cross-sectional | Eastern | Environment | Community members | Drinking from the stream, Eating banku and fufu |
| Osei and Duker,2008 | cross-sectional | Ashanti | Environment | Community members | Proximity to refuse dumps, density of refuse dumps, poor sanitation practices |
| Osei et al.,2011 | cross-sectional | Ashanti | Environment | Community members | Proximity of community to index case, higher population density |
| Osei et al.,2012 | cross-sectional | Ashanti | Environment | Community members | High population density, slum areas, number of refuse dumps |
| Osei and Stein, 2018 | cross-sectional | Ashanti | Environment | Community members | Household interaction, clustering of cases within a radius of 1km, person |
| Osei and Duker,2008 | cross-sectional | Ashanti | Environment | Community members | Urbanization, overcrowding and neighborhood order |
| Eibach et al.,2016 | retrospective | Greater Accra, Central, Western, Eastern, Ashanti, Volta | Hospital | Patients | Old age is a risk factor for mortality |
| Issahaku et al.,2020 |  | Central | Environment | Community members | Visit to the CTC, drinking pipe-borne water, drinking sachet water, eating street vended food, washing hands, poor environmental hygiene |

Supplementary Table 5: Meta Regression

| **Covariates** | **Estimate** | **Standard Error** | **z-value** | **p-value** | **95% CI LB** | **95% CI. UB** |
| --- | --- | --- | --- | --- | --- | --- |
| **Intercept ***** | 0.9512 | 0.8497 | 1.1195 | 0.2629 | -0.7141 | 2.6165 |
|  |  |  |  |  |  |  |
| **Sample** |  |  |  |  |  |  |
| Meat sample | 0.0228 | 0.6947 | 0.0328 | 0.9739 | -1.3388 | 1.3843 |
| Not specified | 0.0757 | 0.6654 | 0.1138 | 0.9094 | -1.2284 | 1.3798 |
| Rectal swab | 0.4383 | 0.6952 | 0.6305 | 0.5284 | -0.9242 | 1.8008 |
| Stool sample | -0.0589 | 0.6946 | -0.0848 | 0.9325 | -1.4202 | 1.3025 |
| Stool sample and rectal swabs | 0.4052 | 0.7775 | 0.5212 | 0.6023 | -1.1187 | 1.9292 |
| Water sample | -0.1510 | 0.6659 | -0.2267 | 0.8206 | -1.4561 | 1.1541 |
| **Region** |  |  |  |  |  |  |
| Central | -0.5013 | 0.8949 | -0.5602 | 0.5754 | -2.2554 | 1.2527 |
| Greater Accra | -0.7813 | 0.6917 | -1.1296 | 0.2587 | -2.1369 | 0.5744 |
| Greater Accra, Ashanti | -0.4592 | 0.9802 | -0.4685 | 0.6394 | -2.3802 | 1.4619 |
| Greater Accra, Central, Western, Eastern, Ashanti, Volta | -0.4463 | 0.6918 | -0.6451 | 0.5189 | -1.8022 | 0.9096 |
| Greater Accra, Northern | -0.7949 | 0.6926 | -1.1477 | 0.2511 | -2.1524 | 0.5626 |
| Northern | -0.4510 | 0.9576 | -0.4709 | 0.6377 | -2.3278 | 1.4259 |
| Not stated | -0.4883 | 0.8935 | -0.5465 | 0.5847 | -2.2396 | 1.2630 |
| Western | -0.4668 | 0.9593 | -0.4866 | 0.6265 | -2.3470 | 1.4133 |

Abbreviations: CI – Confidence Interval; LB – Lower Boundary, UB – Upper Boundary

Supplementary Table 6: Sensitivity Analysis

| **S/N** | **Excluded Study** | **Prevalence (%)** | | **Lower 95% CI** | **Upper 95% CI** | |
| --- | --- | --- | --- | --- | --- | --- |
| 1 | Abana et al., 2021 | 19.65 | | 9.18 | 32.79 | |
| 2 | Abanyie et al., 2025 | 18.98 | | 8.05 | 33.06 | |
| 3 | Acquah et al., 2016 | 17.84 | | 8.05 | 30.34 | |
| 4 | Afum et al., 2022 | 20.27 | | 9.19 | 34.23 | |
| 5 | Akuffo et al., 2017 | 20.29 | | 9.71 | 33.46 | |
| 6 | Baah et al., 2022 | 19.81 | | 9.06 | 33.36 | |
| 7 | Danso et al., 2020 | 16.07 | | 7.45 | 27.09 | |
| 8 | Dzotsi et al., 2015 | 17.56 | | 7.95 | 29.84 | |
| 9 | Eibach et al., 2016 | 18.42 | | 8.39 | 31.16 | |
| 10 | Enchill et al.,2024 | 19.83 | | 9.45 | 32.79 | |
| 11 | Feglo and Sewurah, 2018 | 16.01 | | 6.89 | 27.89 | |
| 12 | Issahaku et al., 2020 | 17.30 | | 7.93 | 29.26 | |
| 13 | Noora et al., 2017 | 15.20 | | 6.93 | 25.85 | |
| 14 | Ocran and Tagoe, 2014 | 17.07 | | 7.53 | 29.37 | |
| 15 | Odonkor and Addo, 2018 | 20.24 | | 9.39 | 33.85 | |
| 16 | Osei-Tutu and Anto, 2016 | | 20.59 | 10.44 | | 33.02 |

Abbreviations: CI – Confidence Interval
